# A systematic umbrella review on the impact of elite sport on participation in physical activity and sport

**DOI:** 10.3389/fspor.2026.1770140

**Published:** 2026-02-10

**Authors:** Vincent Reinke, Jannika M. John, Michael Mutz, Celine Hilpisch, David Jaitner

**Affiliations:** 1 Institute of Pedagogy and Philosophy, German Sport University Cologne, Cologne, Germany; 2 Institute of Sports Science, University of Tübingen, Tübingen, Germany; 3 Institute of Sport Science, Justus Liebig University Giessen, Giessen, Germany

**Keywords:** demonstration effect, high-professional sport, organized sport, physical activity, PRISMA, review of reviews, volunteering

## Abstract

To legitimize the rising public spending on elite sport, governments and political stakeholders frequently refer to the potential beneficial effects of elite sport for society. A frequently given argument centers on the idea that elite sport has a demonstration effect, inspiring people in terms of their participation in physical activity and sport, so that financial support for elite sport will ultimately also reach the grass-roots level. In this systematic umbrella review, we summarize the findings from previous reviews on the actual impact of elite sport on three, partially overlapping dimensions of participation: physical activity, sport, and voluntary work. Following the Preferred Reporting Items for Systematic reviews and Meta-Analyses (PRISMA) 2020 Guidelines, we searched four electronic databases (APA PsycINFO, PubMed/Medline, SPORTDiscus, Web of Science), complemented by a reference and citation screening. Two independent reviewers screened all identified studies for eligibility. Of the 1.005 search results, 13 reviews were included in the final analysis. Their methodological quality was assessed by using an adapted version of the Critical Appraisal Skills Programme (CASP) Checklist for systematic reviews with meta-analysis of observational studies. The synthesis of the reviews’ findings does not allow for any firm conclusions to be made about elite sport’s impact on voluntary work, while a large number of research studies in relation to physical activity and sport, especially in the context of major sport events, can be observed. However, regardless of the conceptual understanding of physical activity or sport, there is only limited evidence for an inherent demonstration effect on various dimensions of participation in physical activity and sport, suggesting that any potential impact of elite sport needs to be leveraged by other supporting activities. Besides highlighting existing findings on relevant success and failure conditions, this umbrella review also elaborates on the significant research gaps that need to be explored in future studies to stimulate a more differentiated and candid debate regarding the role of elite sport for the general participation in physical activity and sport.

**Systematic Review Registration:**

https://doi.org/10.17605/OSF.IO/MKT3Y.

## 1 Introduction

Since the turn of the century, there has been a growing focus on the societal impacts of elite sport. Bids from cities and countries to host elite sport events nowadays routinely include legacy statements detailing how these events should be leveraged to achieve wider societal outcomes. Public expectations and, in some cases, even formal obligations to pursue such goals can be seen as a result of the growing criticism of the increasing national spending on elite sport, especially since respective justifications are perceived as ill-defined and based on overly optimistic assumptions about the benefits to society (1). In the context of societal effects of elite sport, academic and political discussions refer to a wide range of potential benefits (2), with one of the main arguments claiming a positive influence on participation in physical activity and sport when people follow elite sport in many different ways.

For example, the 2012 London Games were the first edition to explicitly define a respective societal goal in advance, specifically, “helping at least two million people in England be more active by 2012” [(3), p. 6]. Furthermore, the initial draft of the German Sportfördergesetz (Sport Promotion Act) claims that “[t]hrough their role-model effect, top athletes are able to inspire their fellow club members and also the entire society to do (more) sport, to join sports clubs, to volunteer or even to participate in competitive sports themselves” [(4), p. 29; translated by the authors]. This line of argumentation can be linked to the notion of a demonstration or trickle-down effect, assuming that the expenditures on elite sport will ultimately reach the grass-roots level, based on the idea that elite sport events, athletes and teams, and their performances inspire the population to increase or (re)take up their participation in physical activity and sport (5).

Research related to elite sports’ impact on participation in physical activity and sport refers to three, partially intersecting contexts: physical activity, sport, and voluntary work. Studies on *physical activity* primarily relate to a bio-mechanical and physiological understanding of physical activity, defined as “any bodily movement produced by skeletal muscles that results in energy expenditure” [(6), p. 126]. Given this strongly epidemiological and health-related approach, such studies argue that physical activities are, depending on their level of intensity, linked to improved cardiovascular and mental health or the prevention of non-communicable diseases and other poor health outcomes for individuals (7), resulting in lower healthcare costs for society (8). Besides this narrow understanding, more holistic approaches to physical activity emphasize both the active role of the individuals and the social embeddedness of physical activity, as it “involves people moving, acting and performing within culturally specific spaces and contexts” [(9), p. 5; for detailed analysis see also (10)]. Regardless of the width of the definition, physical activity manifests itself in various ways, ranging from work, household chores, gardening, or the choice of transportation, up to various movement cultures. The latter includes, for example, attending fitness classes at the gym or jogging in the park. Such activities, however, should not be equated with people's active involvement in the movement culture of *sport*. We refer to the influence of elite sport on people's active involvement in sport following the sociological perspective of Giulianotti (11): Not only is sport culturally situated, underscoring the relevance of power relations, social structures and the diverse meanings, practices and identities attributed to sport by various social groups, it is also ludic, structured, goal-oriented, competitive, thus distinguishing itself from mere exercising practices. Moreover, we attempt to assess the impact of elite sport on participation in sport in terms of *voluntary work*, given its importance for providing others with opportunities to train, play, and compete (12), especially within organized sport, which is discussed as the basis for developing young talents into elite senior athletes (13–15). More generally, both active participation and volunteering in the structures of sport can offer a wide range of opportunities for strengthening the social capital within society (16, 17), while at the same time offering benefits for mental health and well-being (18, 19).

Given these individual and societal benefits, the relationship between elite sport and various forms of participation in physical activity and sport has been the focus of extensive research, including numerous primary studies and several systematic reviews. There is even one “overview of systematic reviews” (20) that, however, only included two reviews, one of which is not peer-reviewed and the other only partially relevant to the research topic at stake. Further, the respective findings are limited to Summer Olympic and Paralympic Games. Against this backdrop, and given the increasing public scrutiny and demand for justification of public elite sport funding over the past decade, there is a three-fold need for a comprehensive synthesis of the existing review literature. To be more precise, this systematic umbrella review synthesizes the key findings of previous reviews on a) the extent to which elite sport impacts participation in physical activity and sport, b) the factors that must be in place for this effect to occur, and c) the research gaps identified by the existing reviews for further studies.

## 2 Methods

This systematic umbrella review has been conducted in accordance with the Preferred Reporting Items for Systematic *reviews* and Meta-Analyses (PRISMA) 2020 Guidelines (21). The research was prospectively registered on the Open Science Framework (OSF) in August 2025 (https://doi.org/10.17605/OSF.IO/MKT3Y).

### 2.1 Database selection and search strategy

Preliminary readings of related articles helped to identify relevant databases, as well as keywords and terms to inform the search strategy. In August 2025, we searched four databases, namely APA PsycINFO, PubMed/Medline, SPORTDiscus, and Web of Science, thereby capturing different research fields, all of which appear relevant to the topic of this umbrella review. The search string covered terms related to both elite sport (as the backdrop for societal effects) and participation in physical activity and sport (as the corresponding outcome), while specifying that the word “review” had to be part of the article's title or abstract. Terms and sets were combined via Boolean operators and carried out using adapted search strings according to the rules of each database (see OSF registration).

### 2.2 Selection criteria and screening process

For inclusion, reviews had to 1) rely at least partially on empirical original studies regarding the effects of elite sport on various forms of participation (i.e., physical activity, sport, and voluntary work); 2) be published in peer-reviewed journals in English or German language since 2000, 3) follow a certain level of systematicity, characterized by using predefined databases and specified search strings. Empirical or conceptual papers were excluded, as were reviews published in books or as grey literature [e.g., (5, 22)]. With regard to peer-reviewed reviews, we did not consider those, which might relate at least partially to the role of elite sport for the general participation in physical activity and sport, but which do not allow conclusions to be drawn about the actual effects, as the result presentation remains at the stage of counting the number of articles published on the respective topic [e.g., (23, 24)]. We also left out systematic reviews focusing on the effects of mass sport events, such as city marathons [e.g., (25)], as well as those studying the impact of volunteering at elite sport events on future intentions to do so in the event sector [e.g., (26)]. The title and abstract screenings were conducted by the first and last author. Discrepancies were clarified through discussion within the whole research team. The first author then performed the full-text screening in consultation with the co-authors, followed by a reference and citation screening using Web of Science.

### 2.3 Data collection and analysis

For each review included, the first author extracted information relevant to our research question. In regular consultation with the second and last author, data were recorded in an Excel spreadsheet, with the following topics constituting the guiding (sub)columns:

Article: bibliographic metadata
Methods of review: review type, languages, time period, methodological guideline, tool for quality appraisal
Content of review: number and types of publications, inclusion & exclusion criteria, conceptualization of participation in physical activity and sport
Objective A: general findings on elite sport's effect on various forms of participation in physical activity and sport
Objective B: conditions for elite sport's effect on various forms of participation in physical activity and sport
Objective C: research gaps and avenues for future research

### 2.4 Quality appraisal

In the absence of a suitable tool for assessing the methodological quality of reviews that use narrative synthesis to report their findings, we adapted the Critical Appraisal Skills Programme (CASP) Checklist for systematic reviews with meta-analysis of observational studies, originally designed to appraise evidence in health sciences (27). We only addressed the checklist questions on the validity and methodological soundness of a systematic review, as we were unable to assess either the trustworthiness of meta-analysis results or their applicability to health interventions in the context of our umbrella review's purpose. The quality appraisal was conducted by the first author. Any concerns were discussed within the whole research team.

## 3 Results

**Figure 1:**
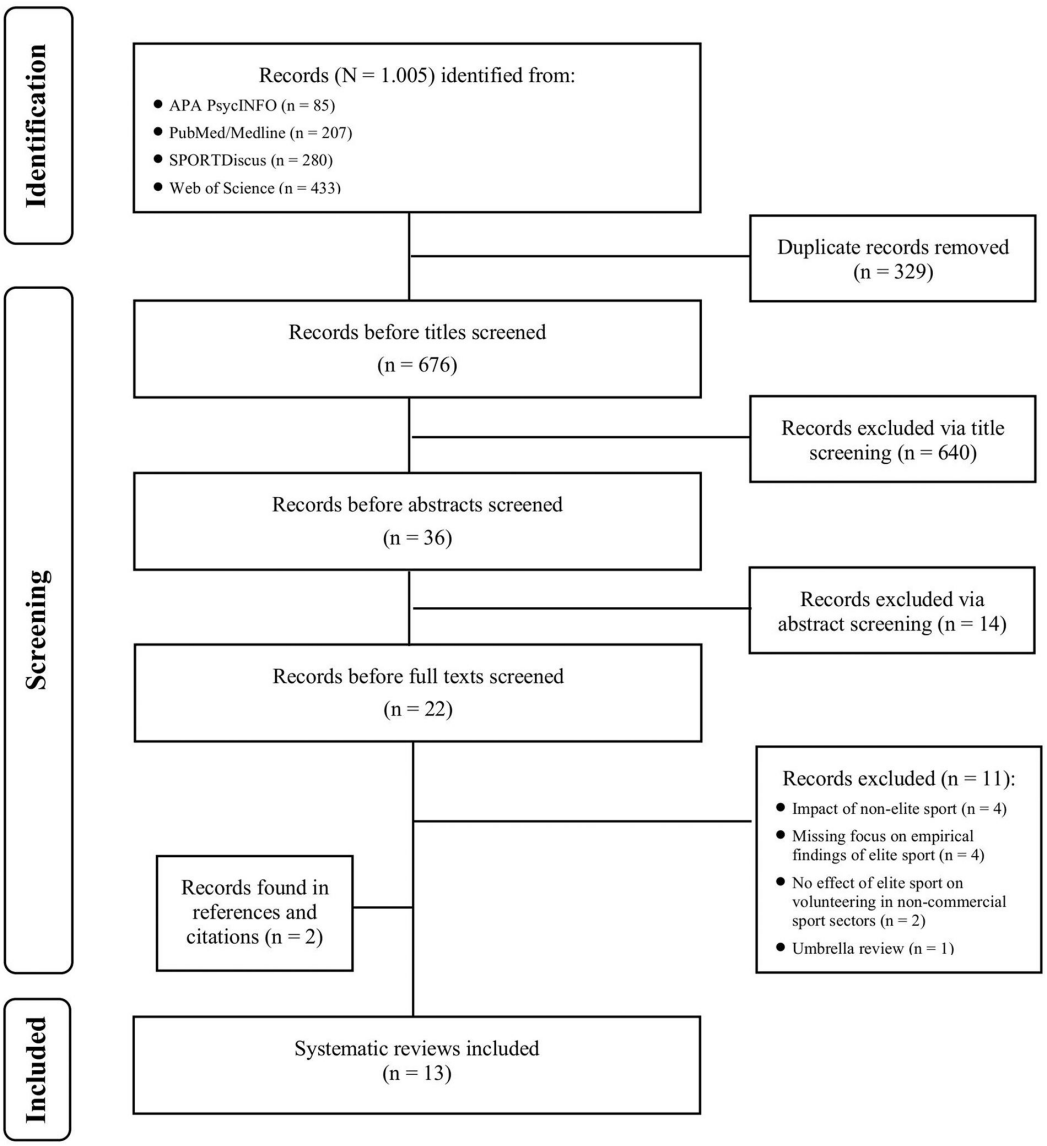
Flowchart of study search and selection process.

The initial literature search yielded 1.005 records. After screening the titles and abstracts, 22 reviews were included in the full-text audit, with eleven articles meeting the inclusion criteria. Two reviews were added through forward and backward citation searches. The search and selection processes are depicted in a flow chart (see Figure 1). Hence, the systematic umbrella review comprises a total of 13 reviews. Seven systematic reviews (28–34), three scoping reviews (35–37), as well as one review using rapid evidence assessment methodology (38) and two literature reviews (39, 40), were considered to fulfill the inclusion criteria.

### 3.1 Descriptive characteristics

According to the characteristics of the included review (see Supplementary Material), more than 30 databases were accessed, with SPORTDiscus and Web of Science (both *n* = 11) and PubMed/Medline (*n* = 10) being by far the most frequently used. Database searches for predominantly peer-reviewed articles were often complemented by reference screening, systematic manual journal, or grey literature searches. According to the inclusion and exclusion criteria defined in the reviews, the role of elite sport for participation in physical activity and sport was addressed almost exclusively on the basis of English-language literature. Eight reviews based their searches on predefined time frames for publications. Five time frames began in 2000, as this was “the first year that legacy plans became a requirement for cities bidding to host the Olympics” [(28), p. 715] and “the public became interested in the legacies of prominent major sporting events” [(30), p. 4]. Remarkably, different reviews highlight the increase of published research on elite sport's impact on participation in physical activity and sport after the 2012 London Olympic Games, which were the first in history to have a clearly stated goal of motivating two million people in society through the event to become more active (36–38, 40).

In view of our research objectives, the reviews, which also include theoretical and conceptual work (33, 34, 36, 37, 40), required us to cross-check the original literature to verify the empirical nature of the results presented. Five reviews focus solely on the effects of elite sport events on different aspects of participation in physical activity and sport (28, 29, 36, 37, 39). Four reviews broaden the perspective by explicitly considering sporting success and the role-model function of elite athletes (often still in the context of sport events) as separate factors influencing participation patterns (31, 33, 34, 38). In line with this event-centered research focus, all reviews that deal with the effects of elite sport on participation in physical activity and sport as only one of several societal aspects do so in the context of sport events (30, 32, 35, 40). None of the included reviews substantially concentrates on the effect of elite sport on volunteering within non-commercial sport contexts, such as in voluntary sport clubs.

In contrast, all included reviews address the impact of elite sport with regard to physical activity and sport, albeit often suffering from terminological vagueness in the use of these key constructs. Only a few reviews provide explicit information on how “physical activity” and “sport (participation)” relate to each other and can be defined and operationalized (28, 31). Some reviews indicate a difference between the two constructs, when they claim to focus on the effect of elite sport on “physical activity (rather than formal sport)” [(34), p. 76] or on “sport participation outcomes [instead of] processes leading to other behavioural outcomes [e.g., physical activity (…)]” [(33), p. 201]. However, the respective authors do not further elaborate on the close relationship between physical activity and sport. Similar shortcomings can be observed in the reviews that examine the effect of elite sport on “sport and physical activity participation” (30, 35), “physical activity and sports participation” (29), or “participation categories […] in terms of sport and/or PA” [(38), p. 2]. Other reviews primarily operate with the term “sport participation”, without clarifying its meaning in relation to the term “physical activity” used in the same manuscript as well (36, 37). Vice versa, other reviews claim to focus on the effect of elite sport on “physical activity”, without specifying the applied understanding, while also employing terms such as “sport(s) participation” in the synthesis and discussion of the empirical evidence (32, 39, 40).

### 3.2 Methodological quality

Four of the 13 reviews do not provide any information on the methodological guidelines used, although they demonstrate a certain degree of systematicity. Within the other nine reviews, the PRISMA guidelines are applied most frequently (*n* = 4), followed by the methodological framework developed by Arksey and O'Malley (41), which is used in all three scoping reviews.

As shown in Table 1, systematic reviews perform best on the CASP questions, which are, though, specifically designed to assess the validity and methodological soundness of exactly this review type. Still, all 13 reviews state clear research questions or objectives and search for appropriate study design(s) to answer them. This also applies to those reviews mentioned above, which are more inclusive in terms of study types, as they also include theoretical and conceptual work on elite sport in connection with participation in physical activity and sport (33, 34, 36, 37, 40). The reviews predominantly report comprehensive search strategies, with the manuscripts providing more or less detailed information about the search terms used. While the reviews are all based solely on English-language publications, they draw on a multidisciplinary range of databases, often supplemented by reference screenings or systematic manual journal searches. Apart from the terminological vagueness mentioned above with regard to “physical activity” and “sport (participation)”, the reviews, more or less explicitly, define inclusion and exclusion criteria in view of their research question(s). Still, some lack precise information on the robustness of the screening process, i.e., whether such screening was carried out independently by at least two people based on the title, abstract, and full-text levels. Similarly, only about half of the reviews contain a flowchart illustrating and summarizing how the final sample of publications was reached, specifying when, why and how many publications were removed.

**Table 1:** Methodological quality of reviews according to adapted CASP checklist for systematic reviews with meta-analysis of observational studies.

Review	1	2	3a	3b	3c	3d	3e	4	5a	5b
Annear et al. (2022) (28)	y	y	y	y	ct	ct	y	y	y	y
Annear et al. (2019) (29)	y	y	y	y	y	y	y	y	ct	y
Inoue et al. (2015) (35)	y	y	y	y	y	y	y	na	y	n
Liang et al. (2024) (30)	y	y	y	y	y	y	y	y	y	y
Lion et al. (2023) (31)	y	y	y	y	y	y	y	y	y	y
McCartney et al. (2010) (32)	y	y	y	y	y	y	y	y	y	y
Murphy and Bauman (2007) (39)	y	ct	y	n	ct	ct	n	n	ct	n
Potwarka and Wicker (2021) (38)	y	y	y	y	ct	ct	n	na	ct	n
Scheu et al. (2021) (40)	y	y	y	y	ct	ct	n	n	ct	n
Shi and Bairner (2022) (37)	y	y	y	y	ct	ct	y	na	y	n
Teare and Taks (2021) (36)	y	y	y	y	y	y	y	na	y	n
Weed et al. (2015) (33)	y	y	y	n	y	y	y	y	ct	n
Weed et al. (2012) (34)	y	y	ct	ct	y	ct	n	y	ct	n

ct, can't tell; y, tyes; n, no; na, not applicable; 1. Did the review address clearly focused research questions? / 2. Did the researchers search for appropriate study design(s) to answer the research question? / 3. Were all the relevant primary research studies likely to have been included in the systematic review? a) Searching for primary research studies; b) Defining inclusion and exclusion criteria; c) Screening primary research studies from the search; d) Selecting primary research studies to include in the systematic review; e) Summarizing the search and its outputs / 4. Did the researchers assess the validity or methodological rigor of the primary research studies included in the systematic review? / 5. Did the researchers extract, and present information from the individual primary research studies appropriately and transparently? a) Extraction of data; b) Presentation of data.

To assess the validity or methodological rigor of the studies, different instruments are used across the included reviews, with the Downs and Black quality index (42) being the only tool applied more than once. Six reviews do not report the use and application of any quality assessment tools, including the scoping reviews. Furthermore, many reviews provide insufficient information on data extraction and do not present key characteristics of the individual studies, such as sample size, time frame, and main results.

### 3.3 The limited evidence for an inherent demonstration effect

The included reviews consistently conclude that there is only limited evidence for a general impact of elite sport on various forms of participation in physical activity and sport in the context of elite sport events (28, 29, 32, 36, 37, 39, 40). Similar findings are also summarized in those reviews that additionally consider the separate effect of sporting success or individual athletes on participation in physical activity and sport, although it proves difficult to draw robust conclusions due to the limited number of original studies in these specific contexts (31, 33, 34, 38). When positive effects on participation patterns are reported, they are described as temporary or related to the period before elite sport events, the so-called ‘pregnancy period’ [(33); see also (36)]. In general, however, the reviews caution against the simplistic expectation that elite sport and related events will inevitably trickle down to the grass-roots level and lead to an increased participation in physical activity and sport, although they do not rule out the possibility of such outcomes. Some reviews, for example, cite single studies suggesting that hosting elite sport events can motivate socially, culturally, or financially disadvantaged groups to try new sports (30) or increase the physical activity among ethnic minorities (37).

### 3.4 Success and failure conditions for increased participation in physical activity and sport

In line with Potwarka and Wicker (38), who conclude that “certain conditions must be met” (p. 8) for an impact on participation in physical activity and sport to occur, we synthesized the success and failure conditions that mediated the influence of elite sport.

#### 3.4.1 The mediating influence of socio-demographic characteristics

Only a few reviews analyze the mediating influence of socio-demographic characteristics in terms of participation in physical activity and sport (30, 31, 36–38). While Liang et al. (30) specifically focus on the influence of major sport events on disadvantaged groups, citing a handful of studies that showed either no effects or small positive effects on participation in physical activity and sport, they do not elaborate on differences in impact compared to more privileged people.

No coherent findings are presented regarding age as a mediating factor. Lion et al. [(31), p. 90] find “no evidence to support an immediate or lagged effect of hosting elite sport events, elite sport success, or elite sport role modeling […] in either young or adult populations”. Both Teare and Take (36) and Shi and Bairner (37) are unable to identify a clear direction of effect, suggesting that the effects vary non-systematically across age groups from one sport event to another. However, Potwarka and Wicker (38) cite several empirical studies according to which the impact of elite sport seems to be more pronounced among youth populations. They indicate that the observed inspirational effect among youth appear to be related to their perception of athletes as role models and elite sport success.

#### 3.4.2 The mediating influence of activity and skill levels among the general public

Some reviews suggest that changes in participation patterns do not necessarily result in more people becoming physically active, for example, through taking up sports. Instead, changed behaviors are mainly found among individuals who already engage in physical activities and sport and possess a positive predisposition towards such participation (29, 33, 34, 36). Weed et al. (33) differentiate between an increase in their participation frequency and a switch in activities by taking up other sports, with the latter finding offering a promising prospect for relatively “new or unusual sports” (p. 210). Their presence at elite sport events may lead to behavioral changes in the form of activity switching among those who already have a positive attitude toward physical activity and sport, and therefore, to greater confidence and readiness to try new pursuits.

In contrast, for inactive people, the high performance level of elite athletes and teams seems to highlight a competence gap in relation to elite athletes so drastically that it potentially has more of a deterrent effect. While elite sport might re-engage lapsed participants (33), inactive people appear to lack confidence in reaching this level of competence, which, in turn, can even result in decreased levels of participation in physical activity or sport (29, 33, 34, 36). Two reviews, however, report some initial findings suggesting that rather inactive individuals may instead be inspired by para athletes (29, 36). Hence, following Annear et al. [(29), p. 679], “a Demonstration Effect may be moderated by the perceived skill level, fitness or functional capacity of the demonstrator in relation to the spectator.”

#### 3.4.3 The relevance of media and live consumption

According to the review of the relationship between sport and population health provided by Inoue et al. (35), the level of personal relevance of professional sport is positively associated with an increase in participation in amateur sport. Closely related, two included reviews highlight the relevance of media or live consumption of elite sport for positive changes in participation in physical activity and sport (36, 38). Potwarka and Wicker (38) emphasize that regular and long-term media coverage of a sport is important for influencing people's participation behavior. According to the author duo, by providing people with the opportunity to enhance their knowledge about a sport, including the athletes and/or teams involved, the media allow people to generate consumption capital that is crucial for forming behavioral intentions to participate in sport after the event. In turn, the lack of media coverage of many sports limits people's ability to accumulate consumption capital in these sports. Hence, Potwarka and Wicker (38) state that “as popular sports are more frequently shown on TV and can, therefore, be watched by many consumers, these sports are more likely to generate [trickle-down-effects] when major events are held” (p. 11)—or, in other words, the influence that different kinds of sports can exert on people's participation in sport depends more on their media reach than their sporting success.

Alongside following elite sport through the media, the affective importance of live spectating is identified as another potential driver for increased participation within included reviews (36, 38): The immediate, intense and immersive experience might help people to enrich their fantasy of being athletes themselves, to fully appreciate the athletes’ efforts and abilities, and to embrace the aesthetics and beauty of the performances and the respective sports. The potentially resulting feelings of inspiration can at least positively affect the formation of a behavioral intention. However, given the lack of empirical insights into how these processes actually translate into changes in participation patterns, “[t]here remains much to be investigated about how spectator events can impact sport participation in terms of whether these impacts exist, to what extent, and under what circumstances” [(36), p. 10].

#### 3.4.4 The idea of an event-related festival effect

The “festival effect”, a term coined in the review by Weed et al. (34), is based on the premise that elite sport events create a festival atmosphere, thereby strengthening people's desire to be part of the collective by joining all kinds of community aspects of the event. Besides spectating or volunteering, mass participation activities organized around elite sport events can also provide such opportunities. In these cases, people's involvement might primarily be intended to experience fun and social connections, rather than necessarily engage in physical activity, but the positive experiences are believed to influence their respective behavioral patterns. While some reviews indicate positive effects of such mass participation activities around elite sport events (29, 34), Shi and Bairner (37) conclude that the festival effect might have “the potential to promote popular sport participation, but there is no evidence to prove this yet” (p. 8).

On the contrary, different reviews refer to the adverse effect that the negative perception of elite sport events can have. Disapproving opinions are linked to more negative attitudes towards and lower rates of physical activity during and after event hosting [(34, 37); see also (29)].

#### 3.4.5 The need for leveraging strategies

In general, the lack of plans to strategically use elite sport, especially in the context of sport events, to increase physical activity and sport among the general population is one of the most frequently given reasons for the limited impact of elite sport on participation in physical activities and sport reported within the included reviews (28, 29, 33, 34, 36–39). The absence of concrete leveraging strategies seems to be at least partly due to the underlying problem that “it is unclear who is responsible for implementing and executing them” [(38), p. 9]. Accordingly, reviews lament the poor coordination and cooperation among all stakeholders, including governments, event organizers, sport federations, sport clubs, and the health sector (29, 37, 39).

In terms of the festival effect mentioned above, Weed et al. (34) demand strategies that use the vibe of elite sport events and people's desire for community by offering enjoyable and social programs for physical activity that engage broad sections of society. Further, several reviews stress the importance of strengthening the infrastructure for physical activities and sport as part of the strategic leverage of elite sport events (28, 30, 37, 39). In this regard, one review emphasizes that facilities used for elite sport events should be made accessible for the public, rather than being abandoned or dismantled afterwards (31). To achieve lasting spillovers from elite sport events, sustained funding is described as essential to support physical activity (29). Financial support is particularly relevant for the education and remuneration of coaches and instructors (38), and, even more fundamentally, for voluntary sport clubs and organizations, given their central role in providing people with opportunities to become or stay physically active (37, 38). More broadly, social, economic, and cultural policies are believed to limit the capacity of organized sport to cater for grass-roots participation that may arise out of elite sport events. Austerity policies in the form of welfare cuts and lower public funding are criticized for eroding the financial resources of grass-roots clubs and organizations, reducing their ability to provide services to the population, which, in turn, negatively impacts individuals’ participation rates (37). Hence, beyond the organized sport, “poor physical activity outcomes may relate to neo-liberal policies of host governments that favor short-term economic outcomes over longer-term public health or social development objectives” [(29), p. 679].

### 3.5 Existing research gaps and avenues for future research

All included reviews offer a more or less critical perspective on existing research gaps. One major research gap relates to the lack of studies on the effect of elite sport on volunteering in organized sport (38). Further, the included reviews call for methodological improvements and suggest avenues for future research.

#### 3.5.1 Defining precise, reliable, and valid constructs

Some included reviews highlight the difficulty of comparing studies since they are based on quite different understandings of key constructs (28, 29, 31, 33, 36, 38, 40). The differentiation between terms like impact, outcome, and legacy is often not given, with these concepts frequently applied interchangeably (36). A similar issue is addressed with regard to the terms of physical activity and sport, “as many studies have not distinguished between these two participation categories” [(38), p. 2]. In terms of physical activity, different reviews criticize the lack of valid and reliable instruments that reflect evidence-based or scientifically sound definitions of the construct (28, 29, 31). According to Scheu et al. (40), this validity and reliability problem is often due to the predominance of studies relying on secondary analyses of quantitative data, which were initially not primarily designed to assess physical activity levels. Moreover, using membership figures as a proxy for individual participation in sport further raises the problem that it is impossible to differentiate between increases in the number of participants, increases in participation frequency, and activity switching (33) or between active individuals and administrative/volunteer members (31).

#### 3.5.2 Improving the study design

Different reviews stress that previous research lacks longitudinal studies to track changes in individual participation patterns and to better understand the causal processes through which elite sport affects participation in physical activity and sport (29, 35, 36, 40). In this context, studies could increase their epistemological value if they were based more profoundly on theoretical foundations to explain the (non-)existence of behavior change between different individuals or groups by considering, for instance, their varying degrees of self-efficacy or resource access, as well as broader interpersonal, organizational, or societal factors (29, 36).

In line with this argument, different reviews criticize original research for often disregarding potential confounders and covariates in their analyses on the effect of elite sport on participation in physical activity and sport, despite the well-known influence of seasonal effects, urban development, traffic, pollution, crime rate (29), socio-demographic characteristics, such as age, gender or income [(28, 31, 38); see also 3.5.3], or social factors, such as friends’ behavior (33). Lion et al. (31) call for more counterfactual analyses, for example, to control for parallel trends of participation in different types of sport, or to compare the effect between the absence and existence of elite sport events or successes.

Such shortcomings could stem from the fact that research on the impact of elite sport on participation in physical activity and sport mainly starts, at least implicitly, from the question of whether there is a positive effect of elite sport. Different reviews point out that, beyond the findings of a deterrent effect in case of a competence gap, the potentially negative impact of elite sport and its athletes on the general participation in physical activity and sport, for example, through sporting failure, doping, or other ethical misconduct, has so far been largely neglected (31, 38).

#### 3.5.3 Diversifying research regarding events, sports, and populations of interest

Despite numerous studies on elite sport events, different reviews identify opportunities to expand the existing evidence concerning the impact on the general participation in physical activity and sport with regard to the event type (28, 36, 37). For example, Teare and Task (36) recognize that the influence of small and medium (spectator) events is significantly under-researched. Also, the existing studies heavily focus on multi-sport instead of single-sport events, making it difficult “to isolate specific legacy effects on population PA, which may be seen more clearly identified in single-sport events” [(28), p. 722]. Among the multi-sport events, the spotlight is very much on Olympic Games, with the potential effect of Paralympic Games being largely ignored (36, 37). Given the above-mentioned preliminary evidence that viewing able-bodied elite athletes may discourage people, regardless of their ability level, while they may be inspired by para athletes, some reviews underline that a focus on para sport could yield relevant insights (29, 36, 37).

Moreover, according to different reviews, studies in the context of elite sport events have so far mainly concentrated on the effects on people in the host country, region, or city, ignoring the global reach of (large-scale) sport events and popular athletes (33, 37, 38). Annear et al. (29) criticize the use of non-representative samples. Another concern across the reviews is the reliance on population-level data, resulting in a lack of in-depth analysis of specific population groups (29, 30, 36–38). Although the value of elite sport is often especially emphasized for youth participation in physical activity and sport, Teare and Taks (36) lament that empirical studies substantiating this claim are scarce. More research is also needed concerning the potential influence of other socio-demographic aspects. For example, Potwarka and Wicker (38) demand not only more empirical knowledge about the potentially differential impact of elite sport on individuals in society depending on their gender (see 3.5.2), but also studies on the potentially differential reach of athletes as role models depending on their gender. Different reviews push for expanding studies on the impact of elite sport on the participation of marginalized groups in physical activity and sport to substantiate the limited evidence on the positive and inclusive role elite sport might play in this context (30, 36, 37). At least partly due to the dearth of corresponding research on the Paralympic Games, there is a lack of evidence on how to change participation rates among people with disability (37).

#### 3.5.4 Providing empirical evidence for the feasibility of leverage strategies

Some reviews also highlight the lack of studies empirically investigating the effectiveness and feasibility of specific leverage strategies (31, 36, 37, 39). So far, research on the respective policy measures has “mostly remained at the hypothesis stage” [(37), p. 10].

Accordingly, whereas the reviews emphasize the importance of strengthening the infrastructure for physical activities and sport, “there have been no analyses of the impact of these environmental changes on subsequent PA participation of host communities” [(39), p. 198]. Moreover, reviews identify a lack of insights on the existence and effectiveness of leveraging strategies aimed at specific sub-populations, such as people with disability (31). One review specifically highlights the need for further research to understand how targeted planning can motivate inactive people who appear deterred by elite sport (36). In general, “[f]uture research should attempt to establish best practices for event leveraging and identify benchmarks for event practitioners to draw from” [(36), p. 11].

## 4 Discussion

Against the backdrop of rising public expenditures on elite sport, this systematic umbrella review summarizes previous reviews on the impact of elite sport on various forms of participation in physical activity and sport, focusing both on the factors that must be in place for such impacts to occur, as well as on the methodological shortcomings of previous research and recommendations for future research.

### 4.1 The demonstration effect as a potential effect

While the included reviews do not provide a systematic overview of empirical knowledge about elite sport's actual impact on grass-roots volunteering, there is still a large body of research reviewed in relation to participation in physical activity and sport, particularly in the context of major sport events. However, apart from the fact that long-term effects could hardly be investigated given the small investigation period set around those events, the reviews provide limited evidence of a genuine short-term demonstration effect of elite sport on physical activity and sport. The idea of a direct causal mechanism, whereby the public funding of elite sport will trickle down to the grass-roots level because elite sport events, athletes and teams and their performances inspire the population to increase or (re)take up their participation in physical activity and sport, must be dismissed as a far too optimistic vision of the supposedly unique influence of elite sport. Instead, this umbrella review follows the conclusion of Weed et al. [(33), p. 211] that “the demonstration effect is more likely to be a potential effect that needs to be leveraged by other supporting activities, rather than an inherent effect.”

Still, the reviews recognize not only the lack of leverage strategies through coordinated collaboration between different stakeholders, but also the lack of studies on existing measures to gather evidence of best practices as benchmarks for the feasibility and effectiveness of future initiatives. When analyzing and developing such strategies, it must be considered that age, individual skill levels in relation to elite performances, the degree of previous participation in physical activity and sport, the form of sport consumption, and the festival atmosphere created through elite sport events could be decisive moderating factors with regard to participation in physical activity and sport among the general population. Moreover, the evaluation and design of leverage strategies would profit from studies that close existing research gaps on elite sport's impact on participation in physical activity and sport. Expanding the scope of research to cover a broader range of events, sports, and population groups, and improving the study design by more thoroughly considering the influence of environmental aspects, social factors, or socio-demographic characteristics beyond age, would lead to a more comprehensive understanding of the conditions necessary for elite sport to contribute to increased participation in physical activity and sport.

### 4.2 Need for more theoretically informed research

Such empirical knowledge must be coupled with more theoretically informed research to better explain how elite sport's influence on getting people active is mediated by processes within and outside the individual, which highlights the value of theories of health behavior change (43). With regard to the individual, two reviews (29, 36) recommend the Transtheoretical Model (TTM), which describes behavior change as a series of stages, with awareness, contemplation, and preparation preceding actual behavior change. The TTM was initially developed in relation to smoking addiction (44), but it could also be used for analyzing the differential effect of elite sport on people's participation in physical activity and sport depending on their previous level. In this context, the review by Weed et al. (33) could inform future research, as they demonstrate the value of the TTM for locating the struggles of elite sport in addressing inactive individuals who are arguably at the start of the process of changing participation patterns.

The self-learning theory by Bandura (45), which posits that individuals learn their behavior by observing and imitating the actions of others, could also strengthen the theoretical foundation of research on the effect of elite sport on participation in physical activity and sport. To be more precise, future studies should expand on initial approaches, which explore the value of Bandura's theory for analyzing the impact of elite athletes as role models in this regard (46–48): Having the theoretical underpinning that learning processes are shaped by the factors of perceiving behavior (attention) and remembering it (retention) could help to shed more light on the relevance of constant exposure to sporting role models, through live spectating, media consumption or mentoring programs, as necessary prerequisites for imitating athletes and changing participation patterns. Moreover, building on the factor of reproduction within the self-learning theory could prompt research to address the relevance of self-efficacy, as a person's belief in their own capabilities and the resulting likelihood to follow the behavior of an elite athlete might primarily depend on the extent to which the individual perceives similarities between themselves and their role models, for example, in age, gender or skill level.

The Social Ecological Model (SEM), mentioned by Annear et al. (29) and Teare and Taks (36), would also allow for a more nuanced theory-driven analysis of the role of elite sport for participation in physical activity and sport, as the SEM account not only for individual factors, but also for the broad range of barriers that prevent certain population groups from translating any potential inspiration from elite sport into actual behavioral change. Originally, the SEM viewed human development as shaped by multiple layers of various systems (49), and was later adapted by Stokols (50) to establish its usefulness in evaluating the effectiveness of (activity-related) health promotion programs with regard to the influences on behavior occurring at multiple levels, covering intra- and interpersonal, environmental, community, and policy factors. Following, with regard to the topic of this umbrella review, the SEM “can be a useful tool for researchers to understand how leveraging initiatives can be designed to stimulate sport participation in a community” [(36), p. 8], taking into account previous findings on the individual, social, organizational, environmental, political, and societal factors that limit the participation in physical activity and sport among marginalized populations (51).

### 4.3 Future research directions

Against this backdrop of exclusionary forces affecting certain population groups, the demand that funding for elite sport must always be closely aligned with the sustainable promotion of organized sport, which is expressed in different reviews (37, 38), appears to be not far-reaching enough. This line of argument for financial support below the top of the sport pyramid needs to be extended insofar as the primary concern must be to make practices and structures in organized sport more inclusive so that participation rates of people from different economic, ethnic, or gender backgrounds can increase (52–54).

Moreover, the reviews emphasize the importance of strengthening infrastructure for physical activity and sport, while they are unable to specify the extent to which improvements to facilities in connection with elite sport events actually have an impact on overall participation patterns (39). In addition to analyzing the relevance of sport facilities, future studies in this context must further elaborate on the ways in which infrastructure programs around elite sport (events) can effectively incorporate ideas of (re-)designing public spaces, such as parks, roads, or squares. Making them more accessible, safer, and greener widens the opportunities for physical activity, especially in the residential areas of marginalized groups (55–57).

Any future analysis and development of leverage strategies should also take into account the potentially negative effects of elite sport. Apart from the competence gap, some reviews point out that elite athletes’ misconduct or lack of success may also contribute to decreasing participation rates (31, 38). Moreover, considering that the individual competence levels in relation to elite athletes and the form of sport consumption seem to be mediating factors, the perception of elite athletes might rely less on their sporting abilities, but rather on the way they present themselves on social media, which “could be used as a platform to inspire physical activity and sport participation” [(58), p. 14]. Such research could encompass comparisons of the role of various types of influencers (e.g., elite athletes, running or fitness influencers). This would allow to assess the differential impact of different potentially relevant inspirational sources on social media users and their participation in physical activity and sport (59, 60).

The above-mentioned lack of a systematic review of elite sport's influence on grass-roots volunteering does not necessarily mean that no studies are available in this regard, as it may simply reflect that existing studies have not yet been systematically reviewed. According to Wicker (12), numerous studies examine individual and structural factors influencing volunteering at the grass-roots level in general, although they lack a nuanced analysis of different volunteer roles, such as coaches or referees. For example, a person's exposure to equivalent actors in elite sport might be more decisive for their decision to volunteer at sport clubs or organizations than following elite athlete performances, as a study indicates regarding the role model function of referees in elite football on the number of referees at the amateur level (61). To draw robust conclusions about the effect of elite sport on volunteering at the grass-roots level, a potential review should also consider papers that refer to the influence of voluntary work around elite sport events on intentions to continue such activities in the non-commercial sport sector (62–64).

### 4.4 Limitations

While this umbrella review critically examines previous research, some limitations of this review itself need to be addressed. Our language filters restricted our search coverage, so the reviews we eventually included refer only to English-language publications. Hence, the scope of the synthesized results is constrained to a predominantly Western, Anglo-American perspective. The findings may also be incomplete, as publication types other than peer-reviewed reviews with a certain level of systematicity were not considered. As a consequence, we not only disregarded a review recently published as a book chapter (22), but this umbrella review also carries the risk of ignoring newer empirical studies that have not yet been synthesized within a review.

Moreover, while our findings address the general debate over whether elite sport contributes to getting more people active, within our results and discussion sections, we have at times struggled to pinpoint and differentiate the effects of elite sport on physical activity from those on sport. The present umbrella review has arguably inherited these shortcomings from the terminological vagueness within the included reviews: They often lack clear conceptualizations of “physical activity” and “sport (participation)”, which, in turn, at least partly seems to result from empirical research not using reliable and valid instruments for the constructs of interest, between which it is difficult to make a distinction due to the conceptual overlap. While this assessment remains at the diagnostic level rather than offering a conceptual solution, there is a need for publications with a strong theoretical and methodological focus to establish a solid framework, within which studies can operate when distinguishing between physical activity and sport.

## 5 Conclusion

Despite the variety of research, this umbrella review shows that empirical evidence on the actual impact of elite sport on various types of participation in physical activity and sport is limited. The unique influence of elite sport appears to be very restricted, with many other individual and societal factors also influencing the participation in physical activity and sport.

Future research must thoroughly analyze the complex interrelationships between different factors potentially affecting the general participation in physical activity and sport to provide new empirical insights for a nuanced and open-ended discussion in academia and society about the societal role of elite sport. Rather than relying on the simplistic notion of an inherent demonstration effect, more theory-driven research on the success and failure conditions is necessary to understand how people's opportunities, resources, and behaviors can be changed and which additional leveraging efforts alongside elite sport are needed to promote an increase in the general participation in physical activity and sport.

## Data Availability

The original contributions presented in the study are included in the article/Supplementary Material, further inquiries can be directed to the corresponding author.
