# Supplementary material for: A systematic umbrella review on the impact of elite sport on participation in physical activity and sport
Source: Front Sports Act Living. 2026 Feb 10;8:1770140. doi: 10.3389/fspor.2026.1770140 (PMC12929462; doi:10.3389/fspor.2026.1770140)
Supplement: Supplementary file 1 [file Table1.docx]

Supplementary Material

Table 1. Descriptive characteristics of the included studies.

| **Source** | **Review type** | **Methodological standards** | **Search strategy, number & time period of publications** | **Inclusion & exclusion criteria** | **Origin** | **Construct** | **Objective A** | **Objective B** | **Objective C** |
| --- | --- | --- | --- | --- | --- | --- | --- | --- | --- |
| Annear et al. (2022) | Systematic review | Guideline: PRISMA  Quality assessment: Downs and Black quality index | Database & grey literature search  12 sources (2011-2018) | In: English peer-reviewed journal articles published between 2000 and 2020, using quantitative studies with experimental, longitudinal, or cohort study design to explore PA related to hosting one of the largest global sport events based on large and representative datasets | Hosting of sport events (largest events, including Olympic Games or World Cups) | PA domains (e.g., sport, exercise or active leisure participation) based on self-reports or direct measurements | Limited effects | Event leveraging | Lack of considering confounders & covariates  Strong focus on (mega) multi-sport events  Validity and reliability problems of PA measures |
| Annear et al. (2019) | Systematic review | Guideline: PRISMA  Quality assessment: Commonly used measures of quality for quantitative and qualitative studies without applying specific tool | Database & grey literature search  9 sources, including 3 reviews (2010-2018) | In: English peer-reviewed journal articles published since the year 2000, constituting primary research or systematic reviews on the capacity of sports mega-events to inspire increases in PA and sports participation among adult populations and on limitations and gaps in existing research  Ex: Book chapters, government reports, and documents from non-government organizations; studies of low methodological quality | Hosting of sport events (mega events, including Olympic and Paralympic Games) | PA and sports participation [not further specified] | Poor evidence | Activity and skill level: deterrent effect due to competence gap  Event leveraging  Festival atmosphere  Perception of the event | Lack of considering confounders & covariates  Lack of long-term/ longitudinal studies  Lack of research on population sub-groups  Lack of theory-driven research  Validity and reliability problems of PA measures |
| Inoue et al. (2015) | Scoping review | Guideline: Arksey’s and O’Malley’s methodological framework, elaborated by Levac et al. | Manual check of literature starting with the references of four earlier reviews & database search  135 sources (1991-2014)* | In: English peer-reviewed journal articles or grey literature (e.g., technical research reports) published between January 1990 and May 2014, constituting empirical studies designed to explicitly examine spectator sport’s influence on a particular aspect of physical, mental, and social well-being, as well as specific health-related behaviors of a population  Ex: Reviews or commentaries, studies examining health variables only as the independent variables, studies on participant sport or sport activities, and studies on the health of elite athletes | Sport events (spectator sport), and athletes (as role models in the context of spectator sport) | Sport and PA participation [not further specified] | Preliminary evidence for the positive short-term impact of spectator services on sport and PA participation |  | Lack of long-term/ longitudinal studies |
| Liang et al. (2024) | Systematic review | Guideline: PRISMA  Quality assessment: Modified version of the QATSDD | Database & manual search (including screening of article references)  14 sources (2011-2023)* | In: English peer-reviewed journal articles published between 2000 and 2023 following the PICOT framework (quantitative, qualitative, or mixed methods studies on outcomes of socioeconomic and health impacts and legacies among non-clinical groups of children and adults from disadvantaged backgrounds experiencing major sport events)  Ex: Articles without empirical data or being irrelevant to the legacies of major sport events for disadvantaged groups | Hosting of sport events (major events, including mega events such as Olympic Games and World Cups) | Sport and PA participation [not further specified] | No or short-term positive impact on sport participation for socially disadvantaged groups | Long-term effects seem to require sustained support and continuous access to sports facilities and programs |  |
| Lion et al. (2023) | Systematic review | Guideline: PRISMA  Quality assessment: Strength of results was evaluated following Taylor et al’s hierarchy of evidence, plus modified version of the Downs and Black quality index | Database & manual search (including screening of article references)  36 sources (2007-2021) | In: English peer-reviewed articles published between January 2000 to August 2021, focusing, entirely or partly, on the effect of elite sport (sport event, athletes’ and teams’ role modelling, sporting success) on PA/sport participation in (segments of) the general population  Ex: non-English articles; book (chapter), (non)government reports, conference proceedings, and working papers; articles without original or with only qualitative data; studies focusing only on perceptions or attitudes regarding PA/sport practice or the effect of elite sport events including mass participation (e.g., marathons) or of incentives (e.g., voucher) for PA participation offered at elite sport events | Hosting of sport events (elite sport events, e.g., Olympic Games), athletes & teams (role model) and performances (sporting success) in the context of elite sport events | PA/sport participation described as the percentage of the population reaching PA recommendations (or another PA practice cut-off), the amount (e.g., minutes) of PA/sport practice or the number of sport memberships (as a proxy for any organized sport participation as a contributing factor to meet PA recommendations) | Null or mixed effects of hosting elite sport events, sport success and role model athletes | Socio-demographic factors (especially age) | Lack of considering confounders & covariates  Lack of research on effectiveness of leverage strategies  Validity and reliability problems of PA measures |
| McCartney et al. (2010) | Systematic review | Guideline: Not reported  Quality assessment:  Modified version of the Hamilton quality assessment tool; an additional set of appraisal questions for qualitative studies based on the approach by Dixon-Woods et al. | Database & grey literature search  54 sources (1984-2009)* | In: literature on the impact on the host population of any one-off, international, multi-sport event focused on a single city/area that took place between January 1978 and January 2008, related to health and socioeconomic impact, including PA, using at least a mix of real and estimated data  Ex: Commentaries without original data or analysis; studies investigating events earlier than 1978, the impacts on visitors, athletes, or spectators, the host population’s support for the event, the non-host population’s opinions about the host area, media portrayals of the event, economic impacts by using exclusively estimated data or simple lists of construction activity related to events | Hosting of sport events (major/ one-off, international, multi-sport events) | PA [not further specified] | Mixed findings |  |  |
| Murphy & Bauman (2007) | Review | Guideline: Not reported  Quality assessment: Not reported | Database & grey literature search  Number & time period of publications not specified | In: Studies on the effect on physical activity resulting from short-term, discrete, and organized elite sport events (e.g., Olympic Games), non-elite mass events, or major population-level health promotion events | Hosting of sport events (large-scale/major, one-off elite sport events)^+^ | PA [not further specified] | Evaluations of major sport events on PA behavior are scarce, showing either a modest or no effect | Event leveraging | Lack of research on the effectiveness of leveraging strategies (improvements in infrastructure, e.g., facilities and finances) |
| Potwarka & Wicker (2021) | Rapid evidence assessment methodology | Guideline: Collins et al.’s guidelines for the production of quick scoping reviews and rapid evidence assessments | Database search  58 sources (1994-2020) | In: English peer-reviewed journal articles with empirical studies examining TDE  Ex: Theoretical investigations, review articles, or project reports | (Hosting of) sport events, athletes (role model) and performances (sporting success) in the context of sport events | Participation in terms of sport and/or PA [differences not further clarified] | TDE may occur, but the effect must not apply to all people in a country | Capacity of community sport  “Epicenter effect”: TDE of a sport event related to the geographic proximity to the hosting city  Event leveraging  Media consumption and live spectating of sport (events)  Socio-demographic factors (especially age) | Lack of considering confounders & covariates (socio-demographic factors)  Lack of research on global influence of sport events (e.g., in hometowns of Olympic medalists or superstars)  Lack of research on negative impact of "role models" or sporting failure  Lack of research on population sub-groups (with regard to gender)  Lack of research on the effect on volunteering in community sport |
| Scheu et al. (2021) | Literature review | Guideline: Weed’s methodological guidelines for review papers  Quality assessment: Not reported | Screening of references of literature from bibliography lists from the Olympic Studies Centre (Lausanne) & database search  322 sources (1984-2017)*^#^ | In: English peer-reviewed journal articles, focusing on the concept of legacy, related methodological issues, and how legacy can be measured in the context of a specific Olympic Games edition (articles using the term impact included if they dealt with structural changes) | Hosting of sport events (Olympic Games) | PA [not further specified] | Olympic Games are often, but not always, considered an effective means to increase physical activity |  | Lack of long-term/ longitudinal studies  Validity and reliability problems of PA measures |
| Shi & Bairner (2022) | Scoping review | Guideline: Arksey and O’Malley’s methodological framework, extended with a systematic manual journal search framework following the guidelines of Teare and Taks | Systematic manual journal & database search  54 sources (2008-2021)^#^ | In: English, online available, peer-reviewed journal articles or book chapters between 2000 and 2021, assessing the impact/legacy of the (Youth) Olympic/Paralympic Games on sport participation | Sport events (Olympic, Paralympic and Youth Olympic Games) | Sport participation [not further specified] | Collecting empirical evidence on the successful use of Olympic sport participation legacies to achieve increased sport participation is still in a lagging phase | Capacity of community sport  Event leveraging  Perception of the event  Socio-demographic factors (especially age) | Lack of research on global influence (e.g., in non-host cities, regions and countries)  Lack of research on the influence of (Paralympic) sport on the sport participation among people with disability  Lack of research on the effectiveness of leveraging strategies, including government policies and the support of community sport |
| Teare & Taks (2021) | Scoping review | Guideline: Arksey’s and O’Malley’s methodological framework, extended with a systematic manual journal search framework following the guidelines of Teare and Taks | Systematic manual journal & database search  146 sources (2004-2018)^#^ | In: English, online available, peer-reviewed journal articles or book chapters published up to and including 2018, addressing impacts, legacies, and leveraging of sport (spectator or participant) events in terms of sport participation  Ex: Sport participation as a potential application (e.g., studies that empirically assessed outcomes of sport events that are not sport participation, but mentioned potential sport participation implications) | Hosting of sport events (spectator events)^+^ | Sport participation [not further specified] | Little or mixed evidence that mega, medium, or small spectator sport events stimulate grass-roots participation  Participation tends to increase in the pregnancy period, i.e., in the run-up to the sport event, but is not sustained | Activity and skill levels: deterrent effect due to competence gap, while active people may participate at higher rates or trying different sports  Live spectating  Event leveraging  Socio-demographic factors (especially age) | Lack of research on population sub-groups  Lack of research on the effectiveness of leveraging strategies  Lack of theory-driven research  No clear-cut definitions of key concepts such as outcome, impact or legacy  Strong focus on mega sport events (without including para sport) |
| Weed et al. (2015) | Systematic review | Guideline: Not reported  Quality assessment: rudimentary approach to the quality appraisal rather than the comprehensive approach to appraising quality of studies normally recommended | Databases & grey literature search  21 sources (1994-2007)^#^ | In: English publications between 1990 and 2008; relevant to the demonstration effect | Sport events (including Olympic Games), teams (franchises) | Sport participation instead of processes leading to other behavioral outcomes (e.g., physical activity [differences not further clarified] | Limited evidence for a (short-term) demonstration effect, especially in the pregnancy period with participation increasing in the lead-up to the sport event | Activity and skill level: deterrent effect due to competence gap; activity switching or increased participation frequency among people already involved in sport  Event leveraging | Lack of considering confounders & covariates  Lack of long-term/ longitudinal studies  Lack of research on global reach of elite sport  Validity and reliability problems of sport participation measures |
| Weed et al. (2012) | Systematic review | Guideline: Not reported  Quality assessment: more rudimentary approach to quality appraisal than is normally recommended for systematic reviews | Database search  24 sources (1991-2007)^#^ | [No access to online information on inclusion & exclusion criteria] | Sport events (including Olympic Games), teams (franchises) | PA (rather than formal sport [differences not further clarified] |  | Activity and skill level: deterrent effect due to competence gap  Festival atmosphere (must be combined with event leveraging)  Perception of the event |  |

Note. * = This figure refers to the number of all sources in the review, although the review only partially deals with the effect of elite sport on participation in physical activity and sport, because the precise number of reviewed sources relevant to participation in physical activity and sport cannot be verified. / ^#^ = This figure refers to the number of all sources in the review, which also includes non-empirical publications. / ^+^ = The review also considered mass participation events, results for those non-elite events were not taken into account here; AMSTAR = A MeaSurement Tool to Assess Systematic Reviews; Objective A = key findings on the extent to which elite sport leads to general participation in physical activity and sport; key findings on the factors that must be in place for the effect of elite sport on participation in physical activity and sport to occur; Objective C = key findings on the research gaps identified for further research; PA = physical activity; PRISMA = Preferred Reporting Items for Systematic reviews and Meta-Analyses; QATSDD = Quality Assessment Tool for Studies with Diverse Designs; TDE = trickle-down effect
